# Overexpressed vascular endothelial growth factor in adipose derived stem cells attenuates fibroblasts and skin injuries by ultraviolet radiation

**DOI:** 10.1042/BSR20190433

**Published:** 2019-07-19

**Authors:** Xiaoyuan Xie, Yina Wang, Yue Xia, Yueping Mao

**Affiliations:** 1 Department of Dermatology, The Third Hospital, Sun Yat-sen University; 2 Special Medical Center, The Third Hospital, Sun Yat-sen University; 3 Department of Dermatology, Sun Yat-sen Memorial Hospital, Sun Yat-sen University

**Keywords:** ADSCs, fibroblasts, photoaging, UV, VEGF

## Abstract

Adipose-derived stem cells (ADSCs) and vascular endothelial growth factor (VEGF) contribute to the healing of wound. The purpose of the present study was to investigate the role of VEGF produced by ADSCs in the protection of fibroblasts and skin of mice from ultraviolet (UV) radiation. ADSCs and fibroblasts were extracted from adipose and skin on the abdomen of mice by enzyme digestion methods. ADSCs surface markers were detected using flow cytometry, and immunofluorescence was used to identify fibroblasts. The expression of VEGF in modified ADSCs with lentivirus was determined. Fibroblasts were injured by UV radiation and co-cultured with ADSCs carrying overexpressed VEGF or normal VEGF. Cell cycle was assessed by flow cytometry. Mice were treated with UV radiation dorsally and injected with ADSCs containing overexpressed VEGF or normal VEGF. mRNA and protein levels of cell senescence-related genes were measured by qPCR and western blot. It was found that ADSCs with overexpressed VEGF not only promoted the effect of ADSCs on down-regulating senescence-associated (SA)-β-Gal, p21 and matrix metalloproteinase (MMP)-1, the healing of wound injured by UV radiation and up-regulating collagen I expression in fibroblasts and wound, but also on inhibiting cell cycle arrest in fibroblasts injured by UV radiation and preventing the skin from photoaging caused by UV radiation. VEGF expression in ADSCs played a key role in protecting skin fibroblasts from ageing, which further allowed the skin to resist photoaging, thereby promoting the recovery of wound injured by UV radiation.

## Introduction

Three main types of UV radiation are characterized in terms of its wavelength as follows: UVA, UVB, and UVC. The UVs that can pass through the ozone layer to the ground are mainly UVA and UVB [1]. It was estimated that about 90% injury of skin was caused by UV radiation, resulting in, for instance, roughness, redness, heat, pain, pigmentation, and telangiectasia of skin, which affect the appearance of face and elicit a series of skin diseases [2,3]. Early in 1990s, it has been proved that in the process of skin repair, fibroblasts were a class of important cells that were almost involved in the entire progress of wound healing [4,5]. The migration and proliferation of dermal fibroblasts, which are the key processes in wound repair, largely affect wound contraction, deposition of extracellular matrix, and reconstruction of tissues [4,5].

The application of mesenchymal stem cells in skin repair has been studied [6,7]. Adipose-derived stem cells (ADSCs) were one type of mesenchymal stem cells that can be easily collected and cultured from excess adipose tissue from human or mouse [8,9]. Research showed that the paracrine effect of ADSCs was particularly significant, as it could secrete a variety of cytokines and growth factors and promote angiogenesis, antiapoptosis and immunization, therefore regulating the cells around them [10,11]. For instance, Yao et al. revealed that the transplantation of ADSCs promoted the healing of skin scald wound of rabbit [12]. Thus, the use of ADSCs could be effective in the treatment of wound healing.

Vascular endothelial growth factor (VEGF), which plays an important role in angiogenesis, acts directly on vascular endothelial cells and therefore promotes the proliferation of blood vessels [13]. When VEGF binds to its receptors, it can up-regulate the expression of, for example, basic fibroblast growth factor (bFGF), VEGF receptor 2, platelet-derived growth factor (PDGF) and promotes the healing of wounds by synergistic action with other cytokines [14]. Therefore, the expression of VEGF in the fibroblasts of the wound edge could reflect the possibility of wound healing to some extent [15].

In view of these facts, we investigated the role of VEGF in the protection of fibroblasts and skin of mice from UV radiation, aiming to provide a novel insight into the treatment of injury caused by UV radiation.

## Materials and methods

### The extraction of ADSCs

The protocol of the present study was approved by the Animal Ethics Board of The Third Hospital, Sun Yat-sen University (approval number SY18050512). Animal experiments were carried out in the laboratory of The Third Hospital, Sun Yat-sen University. Total ten 6-week-old female Kunming mice were purchased from Model Animal Research Center of Nanjing University (Nanjing, China) and maintained in a 25°C atmosphere under 12 h light:12 h dark condition (LD), the mice had free access to food and water. Next, the mice were fixed on an operating table and killed for cervical dislocation. The inguinal skin, which was disinfected with 75% ethanol, was cut by scissors to obtain subcutaneous fat. The surrounding blood vessels and connective tissues were carefully removed. About 10 g of the excess abdominal subcutaneous adipose tissue were extracted and immersed in PBS (Solarbio Life Sciences, Beijing, China) containing penicillin and streptomycin (300 μg/ml) (V900929-100ML, Sigma–Aldrich, MO, U.S.A.) and then rinsed twice with 2 × PBS for 5 min. The tissues were cut into pieces at about 1 mm^3^ and grinded, and the samples were then collected and added with 10 ml of 0.1% type I collagenase (SCR103, Sigma–Aldrich, MO, U.S.A.) in a flask on a shaker and held for 1 h in water bath at 37°C. Collagenase was neutralized by 10 ml of 10% fetal bovine serum (FBS) (10099141, Thermo Fisher, Waltham, MA, U.S.A.) and the samples were centrifuged at 1500 round/min for 10 min at 4°C. The samples were then resuspended with DMEM (10569044, Thermo Fisher, Waltham, MA, U.S.A.) medium and sieved after the upper adipose tissue, and supernatant, which was discarded and further centrifuged at 1500 round/min for 10 min, was then discarded. Then, the samples were resuspended with DMEM and incubated with 5% CO_2_ at 37°C. After 48 h, medium was refreshed and the cells were then observed under a microscope (Olympus IX71, Tokyo, Japan) when the density reached 80–90% in the culture flask.

### The extraction of fibroblasts

Similarly, 10 g of the abdominal skin tissue was extracted and immersed in PBS containing penicillin and streptomycin (300 μg/ml). The skin tissue was digested by DispaseII (17105041, Thermo Fisher, Waltham, MA, U.S.A.) at 4°C overnight, and the epidermis and dermal tissue were separated by forceps on the next day. The samples were cut into pieces using an ophthalmic scissors and placed in a flask containing 10 ml of serum-free DMEM medium with 0.25% trypsin and then incubated in an incubator for 2 h at 37°C in 5% CO_2_. Next the samples were further filtered by a 75 μm filter and centrifuged at 1500 round/min for 10 min. Supernatant was discarded and the cells were inoculated into culture flasks with DMEM medium containing 10% serum, and the medium was refreshed after 48 h. The cells were then observed under a microscope (Olympus IX71, Tokyo, Japan) when the density reached 80–90% in the culture flask.

### The treatment of cells and the set of co-culturing system

A co-culture system for ADSCs and fibroblasts was set by Transwell apparatus (Corning, U.S.A.). The fibroblasts were divided into NC-Fb, Model-Fb, ADSC-Fb, or VEGF-Fb groups. The cells in NC-Fb group were set as negative control (NC) with nontreatment, while the cells in Model-Fb group were exposed to UVA (200 μW/cm^2^/S, UVA lamp, Q-Lab, OH, U.S.A.) for 48 h and incubated in the bottom chamber of a Transwell apparatus supplied with medium. The cells in ADSC-Fb group were exposed to UVA (200 μW/cm^2^/S) for 48 h and incubated in the bottom chamber of a Transwell apparatus supplied with medium and co-cultured with ADSCs in the upper chamber of the Transwell, while the cells in VEGF-Fb group were exposed to UVA (200 μW/cm^2^/S) for 48 h and incubated in the bottom chamber of a Transwell apparatus supplied with medium and co-cultured with ADSCs, which contained overexpressed VEGF in the upper chamber of the Transwell. All cells were carefully cultured for 48 h.

### Immunophenotyping by flow cytometry

About 20 μl antibodies (fluorescent CD29-PE-antibody [order number: 130-102-602, MiltenyiBiotec, BergischGladbach, Germany, 1: 10], CD44-APC-antibody [order number: 130-102-563, MiltenyiBiotec, BergischGladbach, Germany, 1: 10], CD31-FITC-antibody (order number: 130-102-519, MiltenyiBiotec, BergischGladbach, Germany, 1: 10], CD45-PerCP-antibody [order number: 130-102-785, MiltenyiBiotec, BergischGladbach, Germany, 1: 10]) were respectively added to 100 μl cell suspension at a density of 4.5 × 10^7^ cells/ml and incubated in the tubes at 4°C in the dark. The cells were then washed with PBS three times and then centrifuged at 1500 round/min for 5 min. The supernatant was further discarded and 200 μl PBS was added with cells, which were placed on flow cytometry and measured. Cell-Quest software was used to analyze the data.

### Immunofluorescence

1 × 10^6^ cells were fixed with 4% formaldehyde and permeabilized with 0.1% Triton™ X-100 Surfact-Amps™ Detergent Solution (85111, Thermo Fisher, Waltham, MA, U.S.A.) in PBS. The cells were blocked with 1% bovine serum albumin (30036578, Thermo Fisher, Waltham, MA, U.S.A.) in PBS and then incubated with anti-Vimentin antibody (ab92547, Abcam, San Francisco, CA, U.S.A.) at 1:500 dilution and mixed in bovine serum albumin in a refrigerator at 4°C overnight. The next day, secondary antibody, goat anti-rabbit IgG H&L anitibody (ab150077, Abcam, San Francisco, CA, U.S.A.) was diluted to 1:1000 and mixed in bovine serum albumin. The cells were counter-stained with DAPI (ab228549, Abcam, San Francisco, MA, U.S.A.) and diluted in PBST to 2  μg/ml and held for 20  min. The cells were mounted by ProLong™ Glass Antifade Mountant (P36984, Thermo Fisher, Waltham, MA, U.S.A.) and sealed by a cover slip and then visualized under a microscope (Olympus IX71, Tokyo, Japan).

### The assessment of cell cycle by flow cytometry

Cells at different phases of cell cycle were measured by Vybrant™ DyeCycle™ Violet Stain regent (Thermo Fisher, Waltham, U.S.A.) following the protocols. 1 ml cell suspension at a concentration of 1 × 10^6^ cells/ml was prepared in the tubes containing complete medium. 1 μl Vybrant DyeCycle™ Violet stain was added to the tubes and mixed with the cells, which were then incubated at 37°C for 30 min in the dark. Eventually, the cells were analyzed on a flow cytometer under 405-nm excitation and 440-nm emission. Each experiment was performed in triplicate.

### The staining of senescence-associated-β-Gal

The expressed SA-β-Gal in fibroblasts was stained by Senescence β-Galactosidase Staining Kit (#9860, CST, MA, U.S.A.). About 3 × 10^6^ cells were washed with PBS prewarmed three times at 37°C and fixed with 3% formaldehyde at RT for 5 min. The cells were then washed with PBS three times and mixed with freshly prepared SA-β-Gal solution in a flask, which was sealed and placed in an incubator at 37°C for 16 h (the light was blocked). SA-β-Gal solution was then discarded and the cells were carefully washed with PBS and observed under a microscope (Olympus IX71, Tokyo, Japan). The blue deposits in the cells were seen as positive staining of SA-β-Gal.

### The transfection of VEGF by lentivirus

Fibroblasts were divided into control, NC or VEGF groups. The cDNA of VEGF was loaded into lentiviral vectors pLenti6/CMV/V5-DEST (Genepharma, Shanghai, China). About 5 × 10^6^ cells were transferred to a 24-well plate containing 1 ml culture medium. The cells in VEGF group were then added with 500 μl of culture medium and mixed with virus solution at a multiplicity of infection (MOI) of 40. The cells were then mixed with Ploybrene transfection enhancer (No.107689, Sigma–Aldrich, MO, U.S.A., 1:200) and incubated in an incubator at 37°C. The cells in NC group were transfected with vectors carrying NC sequence (synthesized by Genepharma, Shanghai, China), while those in control group were not given any treatment. Total 24 h later, the medium was replaced by fresh culture medium to continue the culturing.

### Quantitative PCR

Following the manufacturer’s instruction, total RNA of cells were extracted by TRIzol (Invitrogen, Waltham, MA, U.S.A.) and transferred to cDNAs using iScript™ cDNA Synthesis Kit (Bio-Rad, CA, U.S.A.) in the same day. The reverse transcription reaction was performed at 42°C for 30 min, followed by a reverse transcriptase inactivation at 85°C for 5 min. The procedures and action system of qPCR were prepared and designed following the instructions of Fast Start Universal SYBR Green Master kit (Roche, Basel, Switzerland) and the gene-specific primers. The reaction system was performed using 2 × SYBER Green master mix (10 μl), cDNA template (1.5 μl), 1 μl forward primer (10 μM), 1 μl reverse primer (10 μM), and ddH_2_O (6.5 μl). Procedures were set as follows: 2 min at 95°C for a hot start, 40 cycles for 15 s at 95°C, 30 s at 60°C, and 60 s at 72°C. The data were normalized to control and analyzed using ^ΔΔ*C*^_T_ method. The primers were listed in Table 1. Each experiment was performed in triplicate.

**Table 1:** The sequences of primers

Primer name	Sequence (5′-3′)
*Glb1* (SA-β-Gal)-forward	ACACTGGCTACCTCCCTCTA
*Glb1* (SA-β-Gal)-reverse	TGGGTGTAGACGGAGGGATA
*P21*-forward	ACAAGAGGCCCAGTACTTCC
*P21*-reverse	AGAAATCTGTCAGGCTGGTCT
*Mmp-1a*-forward	TTACGGCTCATGAACTGGGT
*Mmp-1a*-reverse	GTTGGCTGGATGGGATTTGG
*Col1A1*-forward	CTGGCAAAGACGGACTCAAC
*Col1A1*-reverse	TTCTCTTGAGGTGGCTGAGG
*Vegf*-forward	CATCTTCAAGCCGTCCTGTG
*Vegf*-reverse	GACCCTTTCCCTTTCCTCGA
β*-actin*-forward	TTGTGATGGACTCCGGAGAC
β*-actin*-reverse	TGATGTCACGCACGATTTCC

### Western blot

The cells were lysed by RIPA buffer (89900, Thermo Fisher, Waltham, MA, U.S.A.), and the supernatant was collected after centrifuging the cells at

4°C 16,000 × *g* for 30 min. Total proteins in samples were separated in 10% SDS/PAGE for 90 min at 100 V. Equal amounts (30 μg) of each sample loaded into the PAGE was normalized to β-actin. The membrane was washed in PBST (PBS with 0.2% Tween 20) three times and blocked with 5% nonfat milk for 1 h at RT. Anti-VEGF-A antibody (ab46154, Abcam, San Francisco, CA, U.S.A.), Anti-Glb1-antibody (ab203749, Abcam, San Francisco, CA, U.S.A.), anti-p21 antibody (ab109199, Abcam, San Francisco, CA, U.S.A.), Anti-Collagen I antibody (ab34710, Abcam, San Francisco, CA, U.S.A.), antimatrix MMP-1 antibody (10371-2-AP, PTGlab, PA, U.S.A.) and anti-β actin antibody (ab115777, Abcam, San Francisco, CA, U.S.A.) at a dilution of 1:1000 were mixed with nonfat milk and incubated at 4°C overnight. The membrane was further washed three times in PBST for 5 min and incubated with secondary antibody goat-rabbit IgG H&L (HRP) (ab6721, Abcam, San Francisco, CA, U.S.A.) at a dilution of 1:2000 and mixed with nonfat milk. After 1 h of incubation at RT, the membrane was treated with 200 μl of Pierce™ ECL plus western blotting substrate (Thermo Fisher, Waltham, MA, U.S.A.) for 1 min, and the blots on the membrane were then scanned and filmed in a detector (ChemiDoc MP, Bio-Rad, CA, U.S.A.). ImageJ software (version 1.46; National Institutes of Health, Bethesda, MD, U.S.A.) was used to read the gray level of the blots. Each experiment was performed in triplicate.

### Animal experiment

The animal study was approved by the Ethical Board of The Third Hospital, Sun Yat-sen University. Total 40 6-week-old female Kunming mice were purchased from Model Animal Research Center of Nanjing University. All mice were kept in climate-controlled rooms (at 21°C at 50% humidity) under a 12/12 h light/dark cycle. The animals were given clean water and food and observed daily. During the experiments, the weights of mice were measured and recorded. The hair on the back of the mice was removed using an electric shaving knife, and the exposed skin area was about 4 cm^2^. All the animals were divided into four groups as follows: NC, Model, ADSCs, and VEGF groups. The animals in Model, ADSCs and VEGF groups were irradiated dorsally (2 × 2 cm) by UVA + UVB lamps (Q-Lab, OH, U.S.A.) for 30 days. The intensity of UVA was 6.0 mW/cm^2^, while that of UVB was 0.48 mW/ cm^2^. Radiation was given 1 h a day in the first week, 2 h a day in the second week, 3 h a day in the third week and 4 h a day in the fourth week. The distance from the emitting lamp to the back of the animals was 90 cm. The animals were allowed to move around in the cages during the exposure. 1 × 10^4^ ADSCs were suspended in 100 ml HBSS solution (14025076, Thermo Fisher, Waltham, MA, U.S.A.) and subcutaneously injected into the skin of mice, which were irradiated three times by UVA and UVB in a 7-day interval in ADSCs group. Similarly, 1 × 10^4^ ADSCs with overexpressed VEGF were injected into the mice in VEGF group. The mice in Model and NC groups were given HBSS only. NC group was set as control group.

### HE staining

After receiving 30 days of ultraviolet radiation, the mice in each group were killed for cervical dislocation. Tissues in the wound were collected and fixed with 4% formaldehyde after the mice had been culled and perfused. Next, the tissues were embedded in paraffin and cut into sections, which were loaded to a rack and dewaxed as follows: xylene I: 10 min; xylene II: 10 min; absolute ethanol I: 5 min; absolute ethanol II: 5 min; 95% ethanol: 2 min; 90% ethanol: 2 min; 80% ethanol: 2 min; 70% ethanol: 2 min; distilled water: 2 min; and the procedure of HE staining: hematoxylin stain: 10 min; distilled water: 1 min; 1% acidic alcohol differentiation: 5 s; distilled water: 1 min; 0.2% ammonia: 30 s; distilled water: 1 min; eosin stain: 5 min; distilled water: 30 s. The sections were then dried in a hood and sealed with neutral balsam.

### Statistical analysis

Data were shown as the mean ± S.D. The differences between experimental groups were performed using one-way analysis of variance (ANOVA) followed by Tukey’s test. Statistical analysis was performed using Graphpad 7.0 version. A value of *P*<0.05 was considered as statistically significant. All experiments were performed three times.

## Results

### Cells extracted from abdominal subcutaneous adipose tissues were identified as ADSCs

**Figure 1:**
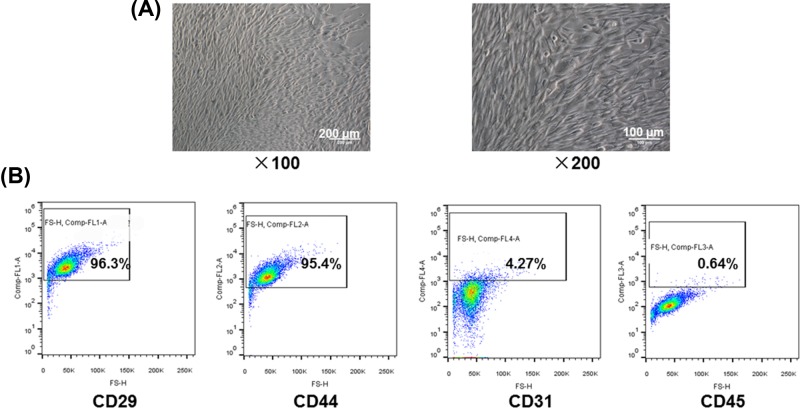
The identification of ADSCs (**A**) The morphology of ADSCs at ×100 and ×200 magnification under a converted microscope (**B**) The immunophenotyping of ADSCs (CD29, CD44, CD31, CD45).

To obtain and identify ADSCs, the cells extracted from abdominal subcutaneous adipose tissues were cultured for 48 h and then observed under an inverted phase contrast microscope, and their immunophenotypings were identified by flow cytometry. As shown in the photographs, we found that the cells were in a flat irregular or polygonal shape with a large protrusion (Figure 1A). In addition, the identification of immunophenotyping by flow cytometry exhibited that these cells expressed a significantly high level of CD29 (96.3%) and CD44 (95.4%), while the CD31 (4.27%) and CD45 (0.64%) were low expressed (Figure 1B), indicating that these cells we extracted from primary tissues and could be seen as ADSCs.

### Cells extracted from abdominal skin tissues were identified as fibroblasts

**Figure 2:**
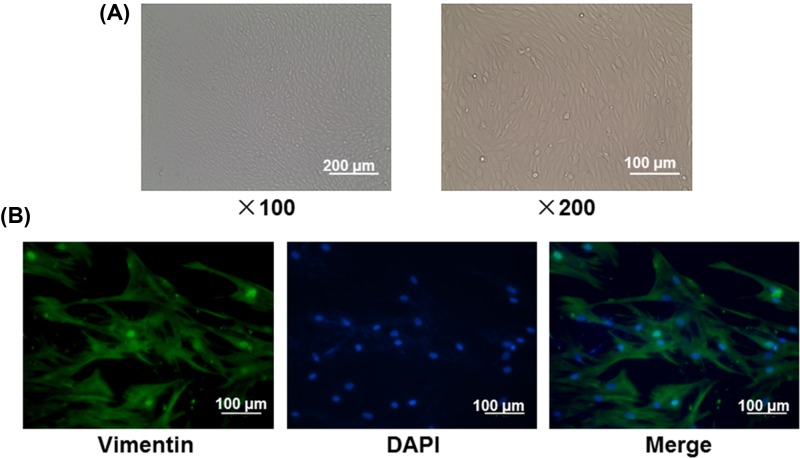
The identification of fibroblasts (**A**) The morphology of fibroblasts at ×100 and ×200 magnification under a light microscope. (**B**) The staining of Vimentin and DAPI in fibroblasts.

To obtain fibroblasts, the cells extracted from abdominal skin tissues were identified by a light microscope and IF. As shown in the images, the cells were in a long fusiform shape or a polygonal shape, which were basically consistent with the shape of ADSCs (Figure 2A). Furthermore, the green fluorescence (Vimentin) was wildly seen in all cells and could be distinguished from DAPI (Figure 2B), indicating that these cells we extracted from primary tissues could be identified as fibroblasts.

### The overexpression of VEGF in ADSCs elevated the effect of ADSCs on protecting the fibroblasts from ageing by UV radiation

**Figure 3:**
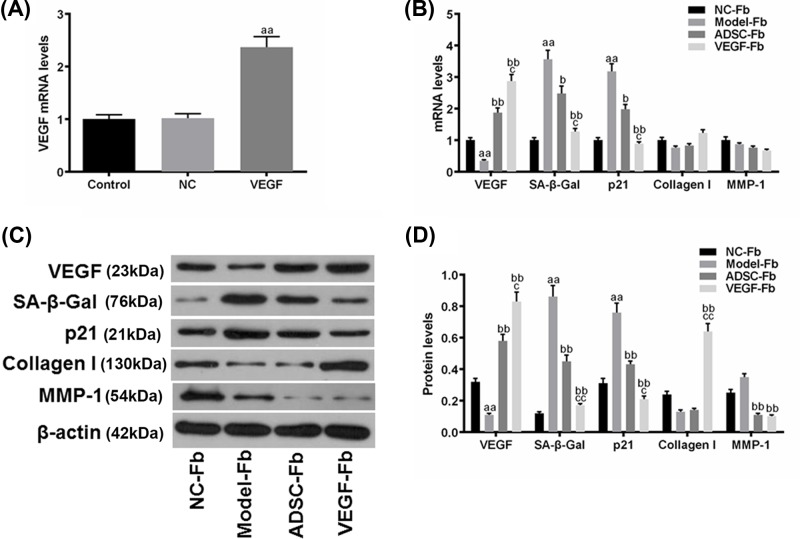
The expressions of VEGF, SA-β-Gal, p21, collagen I and MMP-1 in fibroblasts (**A**) The VEGF mRNA levels in control, NC and VEGF groups (**B**) The mRNA levels of VEGF, SA-β-Gal, p21, collagen I and MMP-1 in NC-Fb, Model-Fb, ADSC-Fb and VEGF-Fb groups (**C**) The original results of western blot (**D**) The protein levels of VEGF, SA-β-Gal, p21, collagen I and MMP-1 in NC-Fb, Model-Fb, ADSC-Fb and VEGF-Fb groups. Bars indicated means ± S.D. of three replicates. ^aa^*P*<0.01 versus NC or NC-Fb group; ^b^*P*<0.05 and ^bb^*P*<0.01 versus Model-Fb group; ^c^*P*<0.05 and ^cc^*P*<0.01 versus ADSC-Fb group.

**Figure 4:**
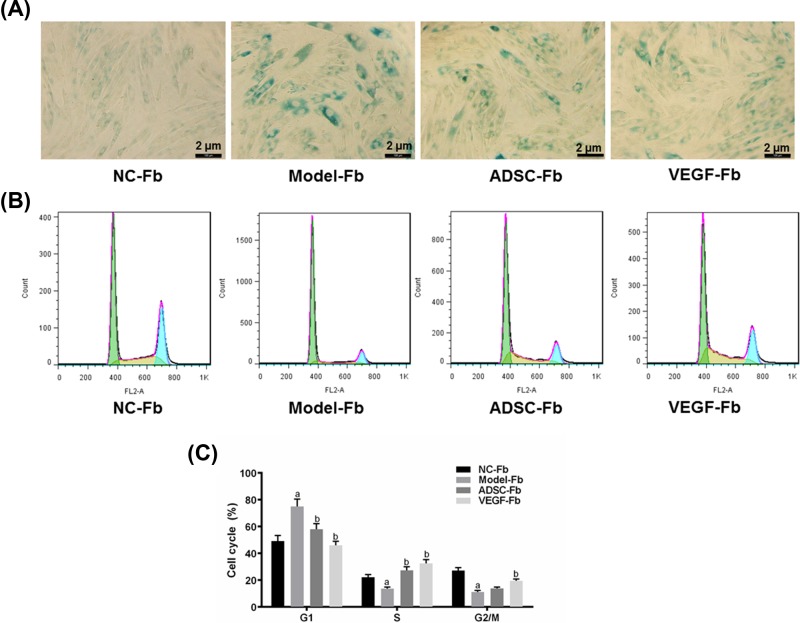
The staining of SA-β-Gal and cell cycle in fibroblasts of NC-Fb, Model-Fb, ADSC-Fb, and VEGF-Fb groups (**A**) The staining of SA-β-Gal in fibroblasts (green) in each group. (**B**) The original diagram showing the number of fibroblasts of each group in different phases of cell cycle. (**C**) The rate of G1, S and G2/M phases of cell cycle in fibroblasts of each group. Bars indicated means ± S.D. of three replicates. ^a^*P*<0.05 versus NC-Fb group; ^b^*P*<0.05 versus Model-Fb group.

In order to investigate whether ADSCs was able to protect the fibroblasts from UV radiation and the role of VEGF in the pathophysiological process of UV radiation, we overexpressed VEGF in ADSCs and measured the expressions of VEGF, SA-β-Gal, p21, collagen I and MMP-1 in fibroblasts that were co-cultured with ADSCs for 48 h. Evidently, VEGF was successfully elevated in ADSCs, compared with NC group (Figure 3A, ^aa^*P*<0.01). Moreover, though the mRNA expression of VEGF in Model-Fb group was significantly lower than that in NC-Fb group, it was higher in ADSC-Fb group than in Model-Fb group (Figure 3B, ^aa^*P*<0.01, ^bb^*P*<0.01). In addition, the expressions of SA-β-Gal and p21 in Model-Fb groups were significantly higher than those in NC-Fb groups, whereas they were lower in ADSC-Fb groups than in Model-Fb groups (Figure 3B, ^aa^*P*<0.01, ^b^*P*<0.05). Interestingly, the expression of VEGF in VEGF-Fb group was sharply higher than that in ADSC-Fb group, by contrast, the expressions of SA-β-Gal and p21 were noticeably down-regulated in VEGF-Fb groups than those in ADSC-Fb groups (Figure 3B, ^c^*P*<0.05). Similar results were observed at the protein levels of these four genes, apart from that the expression of collagen I in VEGF-Fb group was greatly higher than that in Model-Fb and ADSC-Fb groups. Moreover, the protein levels of MMP-1 in ADSC-Fb and VEGF-Fb groups were down-regulated, compared with Model-Fb group (Figure 3C,D, ^aa^*P*<0.01, ^bb^*P*<0.01, ^cc^*P*<0.01, ^c^*P*<0.05). The positive staining of SA-β-Gal in fibroblasts in Model-Fb group was much stronger than that in NC-Fb group, while it was much weaker in ADSC-Fb group than that in Model-Fb group and significantly weaker in VEGF-Fb group than that in ADSC-Fb group (Figure 4A). Taken together, we speculated that VEGF overexpression in ADSCs increased the capacity of ADSCs in preventing fibroblasts from ageing caused by UV radiation.

### The overexpression of VEGF in ADSCs elevated the effect of ADSCs on promoting the cell cycle of fibroblasts injured by UV radiation

To explore whether the cell cycle of fibroblasts could be affected by UV radiation and whether VEGF was involved in the regulation of pathophysiological process of UV radiation, we observed the alterations of cell cycle by flow cytometry. Our data observed that the G1 phase in Model-Fb group was higher than that in NC-Fb group but was lower in ADSC-Fb group than that in Model-Fb group, and G1 phase was much lower in VEGF-Fb group, compared with ADSC-Fb group (Figure 4B,C, ^a^*P*<0.05, ^b^*P*<0.05). Data showed that the S phase in Model-Fb group was lower than in NC-Fb group but higher in ADSC-Fb and VEGF-Fb groups than that in Model-Fb group (Figure 4B,C, ^a^*P*<0.05, ^b^*P*<0.05). In addition, the ratio of G2/M phase in Model-Fb group was lower than in NC-Fb group but higher in VEGF-Fb group than that in Model-Fb group (Figure 4B,C, ^a^*P*<0.05, ^b^*P*<0.05). Thus, it suggested that the cell cycle of fibroblasts was arrested in G1 phase after the cells had been injured by UV radiation, and that the proliferation of fibroblasts was inhibited. However, ADSC promoted the cell cycle of fibroblast, which could be further enhanced by the overexpression of VEGF in ADSC.

### The overexpression of VEGF in ADSCs elevated the effect of ADSCs on promoting the healing of wound injured by UV radiation

**Figure 5:**
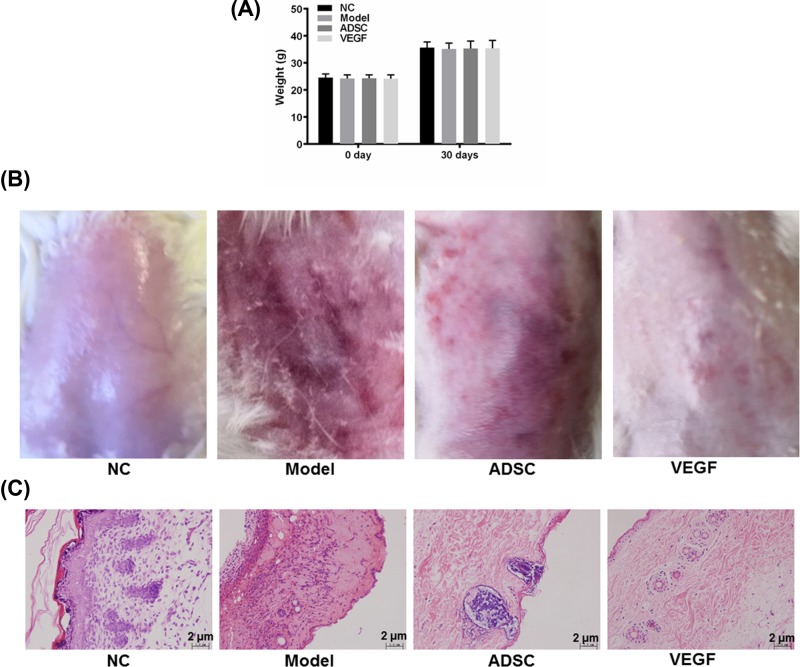
The weight of mice and skin condition in NC, Model, ADSC and VEGF groups (**A**) The weight of mice after 0 and 30 days since the experiments in each group. (**B**) The gross appearances of skin damaged by photoaging in each group. (**C**) The HE staining of skin tissue damaged by photoaging in each group. Bars indicated means ± S.D. of three replicates.

As VEGF produced an important effect on protecting fibroblasts from UV radiation, we further explored the effect of VEGF *in vivo*. As presented in the results, no significant difference of the weight of mice amongst NC, Model, ADSC and VEGF groups were identified after 30 days of radiation injury (Figure 5A). Remarkably, in the Model group, the skins of mice were rough and had deep wrinkles, pigmentation and erythema (Figure 5B). The results of HE staining also revealed that the thickness of the epidermis of the mice increased significantly, while the thickness of the dermis decreased sharply and the collagen fibers increased abnormally in a disordered arrangement, showing the characteristics of an early stage of skin ageing (Figure 5C). Moreover, the skin condition in ADSC group was improved, as the skin was smoother with less wrinkles, pigmentation or erythema than Model group (Figure 5B). However, the accumulations of basophilic substances were observed in the epidermis (Figure 5C). Noticeably, the skin condition in VEGF group was much better than that in ADSC group, as the skin in the former group was nearly recovered from the injury caused by UV radiation and was similar to the condition in NC group (Figure 5B). The results of HE staining also observed neovascularization in epidermis (Figure 5C). Therefore, our data indicated that the overexpression of VEGF in ADSCs could significantly promote the healing of wound injured caused by UV radiation.

### The overexpression of VEGF in ADSCs elevated the effect of ADSCs on preventing the skin from photoaging due to UV radiation

**Figure 6:**
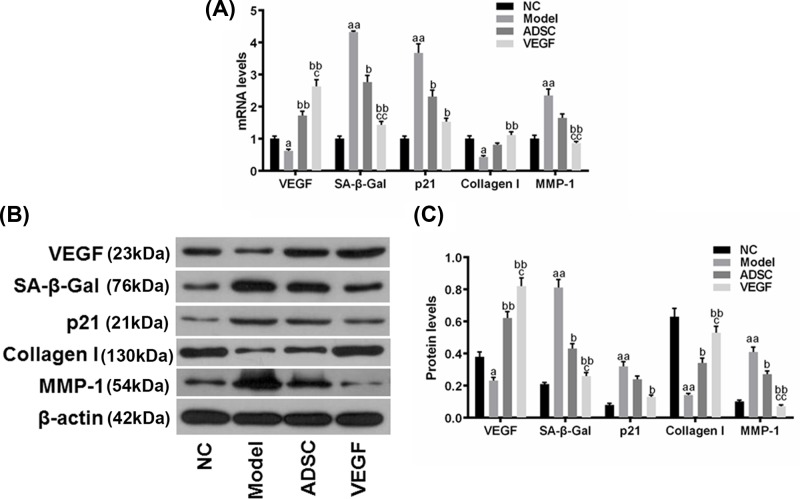
The expressions of VEGF, SA-β-Gal, p21, collagen I, and MMP-1 in skin tissue damaged by photoaging in NC, Model, ADSC and VEGF groups (**A**) The mRNA levels of VEGF, SA-β-Gal, p21, collagen I and MMP-1 in each group (**B**) The original results of western blot (**C**) The protein levels of VEGF, SA-β-Gal, p21, collagen I, and MMP-1 in each group. Bars indicated means ± S.D. of three replicates. ^a^*P*<0.05 and ^aa^*P*<0.01 versus NC group; ^b^*P*<0.05 and ^bb^*P*<0.01 versus Model group; ^c^*P*<0.05 and ^cc^*P*<0.01 versus ADSC group.

We measured the expressions of VEGF, SA-β-Gal, p21, collagen I, and MMP-1 in the skin tissue to assess the decrepitude of the skin, and found that the mRNA expression of VEGF in Model group was significantly lower than in NC group but higher in ADSC group than Model group; noticeably, the mRNA expression of VEGF was much higher in VEGF group than that in ADSC group (Figure 6A, ^a^*P<*0.05, ^c^*P<*0.05, ^bb^*P<*0.01). Moreover, the expression of SA-β-Gal in Model group was higher than in NC group but lower in ADSC group than that in Model group, while it was even much lower in VEGF group than that in ADSC group (Figure 6A, ^aa^*P*<0.01, ^bb^*P*<0.01, ^cc^*P*<0.01, ^b^*P*<0.05). Furthermore, the expression of p21 in Model group was higher than in NC group but lower in ADSC and VEGF groups than that in Model group (Figure 6A, ^aa^*P*<0.01, ^b^*P<*0.05). In addition, the expression of collagen I decreased in Model group, compared with NC group; however, its expression was still higher in VEGF group than that in Model group (Figure 6A, ^bb^*P*<0.01, ^a^*P<*0.05). Though the expression of MMP-1 in Model group was significantly higher than that in NC group, it was lower in VEGF group than that in Model and ADSC groups (Figure 6A, ^aa^*P*<0.01, ^bb^*P<*0.01, ^cc^*P*<0.01). Similar outcomes were observed at the protein levels of these molecules (Figure 6B,C, ^aa^*P*<0.01, ^bb^*P*<0.01, ^cc^*P*<0.01, ^a^*P*<0.05, ^b^*P*<0.05, ^c^*P*<0.05). The data suggested that the photoaging of skin caused by UV radiation was evidently depressed by the overexpression of VEGF in ADSCs, and such an effect became stronger than using ADSCs alone.

## Discussion

In the present study, we extracted and identified ADSCs and fibroblasts from mice, and found that VEGF played a critical role in the protection of fibroblasts and skins of mice from UV radiation. Our results provided evidences showing the effectiveness of modification of ADSC, especially the modulation of VEGF, in the treatment of injury caused by UV radiation.

Here, on one hand, we found that the extracted cells from adipose tissues of mice were in a flat irregular or polygonal shape with a large protrusion after 48 h of culturing. Such a cell behavior was consistent with a previous report, which indicated that these cells were ADSCs [16]. On the other hand, we found that the expressions of CD29 and CD44 were at high levels, while those of CD31 and CD45 were low, which was also consistent with the outcomes of the immunophenotyping of ADSCs from previous research [17,18]. Taken together, we confirmed that the extracted cells from adipose tissues of mice were ADSCs.

Further, we extracted fibroblasts from mice, and found that cells were also in a long fusiform or a polygonal shape, which assembled the shape of ADSCs, and our results were in accordance with a previous study [19]. Vimentin has been accepted as a common molecular marker for fibroblasts [20]. We found that the cells we extracted could be stained positively by Vimentin antibody, confirming that the cells were skin fibroblasts.

Cellular senescence is a basic cellular response after cells has been subjected to specific stimuli [21]. Normally, cells lose their ability to proliferate during the progress of senescence; however, they can still survive for several weeks [21]. SA-β-Gal is a hydrolase that catalyzes the hydrolysis of β-galactoside to monosaccharides and is a long-established reliable marker in detecting cellular senescence [22]. p21 protein is an inhibitory protein of the intracellular cyclic regulatory protein, namely, cyclin E/cyclin-dependent kinase (Cdk) 2 complex, which plays a negative role in the regulation of the cell cycle [23]. In addition, p21, which is an important regulator of cellular senescence, is activated under the regulation of p53 protein and is considered to be an important factor linking p53 and Rb, and therefore p21 could cause cell senescence [24,25]. Collagen I and collagen III are the main components in maintaining the strength and elasticity of the skin [26]. The richness of skin collagen reflects the decrepitude of the skin or cells [26]. Study has shown that UV radiation could increase the activity of MMPs and induce the expressions of MMP-1, MMP-3, and MMP-9 in normal human epidermis, and that MMP-1 can degrade collagen I and collagen III, destroying the formation of skin [27]. In addition, UV radiation also allows keratinocytes to release cytokines and then indirectly promotes fibroblasts to express MMP-1 in a paracrine manner [28]. Evidently, we found that the co-culturing of ADSCs and fibroblasts could increase the expression of VEGF and decrease the expressions of SA-β-Gal, p21 and MMP-1 in fibroblasts, compared with the cells in NC group. Surprisingly, the overexpression of VEGF in ADSCs enhanced such an effect of ADSCs and even significantly elevated the level of collagen I. Compared with Model-Fb group, the positive staining of SA-β-Gal was decreased in fibroblasts co-cultured with ADSCs, and such a decrease was more significant in fibroblasts co-cultured with ADSCs under the effects of overexpressed VEGF. These results indicated that the cellular senescence of fibroblasts was elicited due to the exposure of UV radiation, and that ADSCs could attenuate such damage to some extent. However, the overexpression of VEGF in ADSCs could further strengthen such an effect. Wang et al. reported that ADSCs had protect human dermal fibroblasts from ageing caused by the exposure of UVB [29]. Moreover, evidence had shown that the expression of VEGF receptor-1 (VEGFR-1) in human corneal fibroblast was decreased with ageing [30]. In addition, Chen et al. revealed that mesenchymal stem cells could attenuate doxorubicin-induced cellular senescence via VEGF pathway in cardiomyocytes [31]. Thus, we speculated that the expression of VEGF was critical in protecting fibroblasts from ageing caused by UV radiation, and that the protective effect of ADSCs on fibroblasts could be explained by that ADSCs could produce a considerable amount of VEGF and therefore exhibited such effect [10].

During the ageing, cells will exhibit specific ageing characteristics, and the main hallmark of them is the loss of ability to go through all phases of a cell cycle [32]. Senescent cells are usually arrested in the G0 or G1 phase of the cell cycle; however, they still have metabolic activity [32]. We found that ADSCs was able to promote the cell cycle of fibroblasts and then lead to the down-regulation of G1 phase and up-regulation of S phase; however, the overexpression of VEGF in ADSCs improved such effects of ADSCs and even raised the ratio of G2/M. Kim et al. reported that ADSC-conditioned medium (CM) reduced sub-G1 phase of dermal fibroblasts (HDF) [33]. Knizetova et al. revealed that the autocrine regulation of glioblastoma cell cycle was realized by VEGF-VEGFR2 interplay [34]. This suggested that the proliferation of fibroblasts was suppressed due to the interference of cell cycle caused by UV radiation, and this progress could be reduced by the production of VEGF in ADSCs.

Here, as demonstrated in the figures, we showed the representative photographs of wound damaged by UV radiation, and found that using ADSCs in the wound on mice could evidently heal such damage, though the skin in the wound area was still tough with pigmentation and erythema and basophilic substances was observed by HE staining as well. Nevertheless, using ADSCs in combination with overexpressed VEGF demonstrated a better outcome; however, the weight of mice were not affected. Previously, researchers discovered that the injection of ADSC in the wound significantly increased dermal thickness and the amount of fibroblast in hairless mice [35]. Furthermore, Philips et al. revealed that ability of antiskin ageing of copper was realized through its stimulation of VEGF at physiological concentrations [36]. In addition, Gunin et al. demonstrated that age-related decrease of dermal blood vessels could be seen as a consequence of the impairment of VEGF signaling [37]. Therefore, we speculated that the expression of VEGF in ADSCs might play an important role in the recovery of wound caused by UV radiation.

UVA and UVB radiation could damage the DNA and further lead to the elevation of MMPs, which are able to degrade collagens as aforementioned and elicit the injury of skin, which is known as photoaging [38]. We observed the up-regulations of VEGF and collagen I and down-regulations of SA-β-Gal, p21 and MMP-1 in ADSCs, which were even more noticeable in ADSCs with overexpressed VEGF, and such a result indicated that photoaging was shown on the skin damaged by UV radiation. However, such a trend could be reversed by the overexpression of VEGF in ADSCs, and this might be explained by the possibility that the overexpressed VEGF in ADSCs not only prevented the fibroblasts in skin from ageing, but also promoted their proliferation and therefore granted the skin the ability to resist the photoaging caused by UV radiation. In addition, the promotion of overexpression of VEGF to the neovascularization in local skin could be another possibility, which showed the importance of VEGF in protecting skin from photoaging.

## Conclusion

In conclusion, the expression of VEGF in ADSCs played a key role in protecting skin fibroblasts from ageing caused by UV radiation, which further allowed the skin to be able to resist photoaging; therefore, the recovery of wound injured by UV radiation could be promoted.

## Abbreviations

ADSC: adipose-derived stem cell
MMP: matrix metalloproteinase
NC: negative control
SA: senescence-associated
UV: ultraviolet
VEGF: vascular endothelial growth factor
